# Phytochemicals, Antioxidant and Antiproliferative Properties of *Rosmarinus officinalis* L on U937 and CaCo-2 Cells

**Published:** 2017

**Authors:** Yacine Amar, Boumediene Meddah, Irene Bonacorsi, Gregorio Costa, Gaetana Pezzino, Antonina Saija, Mariateresa Cristani, Soulef Boussahel, Guido Ferlazzo, Aicha Tirtouil Meddah

**Affiliations:** a *Laboratory of Bioconversion, Engineering and Microbiological Food Safety, University of Mascara (29000), Algeria.*; b *Laboratoryof Immunology and Biotherapy, Department of Human Pathology University of Messina, 98125- Messina, Italy.*; c *Department of Pharmaco-chemistry, University of Messina, Viale Annunziata, 98168 - Messina, Italy.*

**Keywords:** Antioxidant activity, Antiproliferative activity, Apoptosis, Cell cycle, Rosemary extracts

## Abstract

*Rosmarinus officinalis *L., a medicinal herb from the labiates family, has been reported to have potential benefit in the treatment and prevention of several diseases. In particular its phenolics have demonstrated protective effects on various types of cancer through several mechanisms. The present study aimed to determine the effects of rosemary phenolic extracts on human cell functions, with particular regard to their anti-proliferative properties in three cell types U937, CaCo-2 and the peripheral blood mononuclear cells (PBMCs). The radical scavenging and Ferric reducing abilities of the extracts have been assessed as well as their cyto-toxicity and effects on cell cycle distribution and apoptosis. About 13 compounds were identified with dominance of rosmarinic acid in the methanolic extract and phenolic diterpens in the ethyl acetate fraction (Carnosol, Carnosic acid and methyl Carnosate). The total polyphenolic content was important in the first extract with 2.589 ± 0.005 g/100 g in gallic acid equivalent compared to 0.763 ± 0.005 g/100 g. The methanolic fraction displayed higher antioxidant activity (DPPH_IC50_: 0.510 mg/mL and FRAP: 1.714 ± 0.068 mmol Fe^2+^/g) while ethyl acetate showed pronounced antiproliferative effects (IC_50_: 14.85 ± 0.20µg/mL and 14.95 ± 2.32 µg/mL respectively for U937 and CaCo-2 cells). The anti-proliferative effect was associated with a cell cycle arrest in S phase for U937 (62% of the population at 5 µg/mL) with a concomitant decrease in G1 and G2/M phases. Tested extracts displayed in addition early apoptotic effects in U937 and late apoptosis in CaCo-2 cells. The obtained data indicate that the identified phenolics are at least partially responsible for the observed cytotoxicity.

## Introduction

In light of the continuing need for effective anticancer agents and the association of fruit and vegetable consumption with reduced cancer risks, edible plants are increasingly considered as sources of anticancer drugs (1). In last decades a great number of dietary phytochemicals including polyphenols like ﬂavonoids, hydrolysable, and condensed tannins, have been studied and characterized for their biological activities (2, 3). In particular, polyphenols received a great part of interest favor to their wide diversity with more than 8000 different compounds described so far (4). Polyphenols have been shown to exert anticancer effects via mechanisms that include antioxidant, antiproliferative, and anti-inﬂammatory activities as well as their effects on intracellular signaling pathways that might implicate apoptosis, and cell-cycle arrest (3, 5). They constitute therefore a promising source of potential chemotherapeutic compounds (6, 7). Rosemary (*Rosmarinus ofﬁcinalis *L.), a culinary spice and medicinal herb, has been widely used in folk medicine to treat numerous ailments (8). Traditionally, it was used for relieving renal colic, asthma, spasmogenic disorders, peptic ulcer, inflammatory diseases, hepatotoxicity, atherosclerosis, ischaemic heart disease, cataract, and poor sperm motility (9, 10). A population-based study has suggested that an overall risk reduction in cancer incidence is correlated with patients consuming herbs including rosemary (11). In particular its phenolic constituents have been found to exert protective effects on various types of cancer; however the mechanisms underlying the observed effects are not hitherto put at light (12). Herein, we intend to further evaluate the antiproliferative and antioxidant potentials of two phenolic-enriched extracts obtained from *Rosmarinus officinalis* L spontaneously growing in north Algeria as a possible source of cytotoxic compounds. 

## Materiel and Methods

*Chemicals*

2,2-Diphenyl-1-picrylhydrazyl (DPPH) was supplied by Sigma Chemicals Co., MO, St Louis, USA, 2,4,6-tripyridyl-s-triazine (TPTZ) and ferric chloride.6H2O were purchased from Sigma Aldrich s.r.l.(Milan, Italy). Paclitaxel from Bristol-Myers Squibb (Princeton, USA), Dimethyl sulfoxide (DMSO) from Sigma Aldrich (France) and Trypan blue 0.4% from McLean (Virginia, USA). L-Glutamine was provided by Euro-Clone, Italy while Penicilline /Streptomycine and bovine foetal serum were purchased from Gibco for life technologies (Grand Island, USA). 

*Extraction of phenolics from Rosmarinus officinalis L.*

The plant of interest consists of a north Algerian chemotype of *Rosmarinus officinalis *L., harvested in the period from March to April. The extraction procedure has been realized for dry leaves by adding three crud solvent respectively hexane extract (HE), Ethyl Acetate (EA) and Methanol (MeOH). The dry matter has been stirred with 400 mL of the first solvent (used here only to remove the lipidic phase) under magnetic rotation during 8 h and then filtrated by Watman paper. The operation was repeated two times more, and then the residual *Rosmarinus* (pellet) is sequentially extracted in the same way by the other respective solvent to yield the crude Hexane, Ethyl acetate and MeOH extracts. The two latest extracts were dried by Rota-evaporation, solubilised in DMSO and then kept in the dark at -30 °C until their use (5). 

*Chemical characterisation*

*Determination of total phenolic content:*

The antioxidant capacity (expressed as content of total phenols) of the *Rosmarinus officinalis *L. extracts was determined by means of the Folin- Ciocalteau reagent (13). 50 µL of methanol/water solutions containing different concentrations of the extracts to be tested were added with 450 µL of deionized water, 500 µL of Folin-Ciocalteau reagent and 500 µL of 10% aqueous sodium carbonate solution; then samples were maintained at room temperature for 1 h. Absorbance was recorded at 786 nm (UV-Vis Spectrophotometer, Shimadzu Japan) against the blank containing 50 µL of the same solvent used to dissolve the extracts. Total phenol content was expressed as g of gallic acid equivalents/100 g extract, using calibration curves prepared with gallic acid standard solutions. Each determination was performed in duplicate and repeated at least two times.

*Identification of phenolic compounds*

Enriched Phenolic extracts from *Rosmarinus officinalis* L. were analysed using high performance liquid chromatography (HPLC) equipment (14). The system was equipped as follows: two SCL-10-AVP pumps, an SCL-10-AVP controller, a photodiode array detector (SPD-M10 Avp), a DGU-14A degasser (all the equipment was Shimadzu); the column used was a C18 (Supelco, Milan, Italy), 250 × 4.6 mm i.d., 2.7 μM particle size. The binary mobile phase consisted of water (A) and acetonitrile (B) both acidified with acetic acid. The gradient was: 0-5 min (2% B), 5-120 min (2-100% B). All the solvents were HPLC grade (Merck, Germany). Volume injected was 20 μL at a flow rate of 1 mL/min. UV spectra were acquired from 190 to 370 nm, and the chromatogram was extracted at 280 nm. At the end, an injection was done on liquid chromatography coupled to mass detection (LC-MS) in order to identify the different polyphenols.

*Cell lines and culture conditions*

The human colon cancer cell line Caco-2 (adenocarcinoma, ^®^^™^) was grown in DMEM medium (Lonza, Belgium) supplemented, with 10% (v/v) fetal bovine serum, 1% (v/v) nonessential amino acids, 1% (v/v) L-glutamine and 1% (v/v) antibiotic solution (Penicillin/streptomycin). The U937 cell line (Human immortalized macrophage, ATCC: CRL 1593) and PBMCs (peripheral blood mononuclear cells) were grown in RPMI 1640 medium (Euro-lone, Italy) supplemented as cited without adding the amino acids. The PBMCs were obtained from whole blood of five healthy adult volunteers without any pathology or treatment (blood transfusion service, polyclinic, Messina, Italy) using Ficoll methode of separation (Cedarlane, Netherlands).

*Antioxidant activity*

*Radical scavenging capacity (DPPH assay)*

The free radical-scavenging capacity of *Rosmarinus officinalis *L. extracts was tested as bleaching of the stable radical DPPH (15). The reaction mixture (1.5 mL of methanol) contained 100 mM DPPH• and 37.5 µL of extracts tested at different concentrations (0.25, 0.5, 1 and 2 mg/mL), an equal volume of the solvent employed to dissolve the extracts (DMSO) was added to control tubes. After 20 min at room temperature, the absorbance was recorded at 517 nm in a UV-VISIBLE spectrophotometer (Shimadzu, Japan). The percentage of inhibition was calculated using the following equation:

%DPPH _remaining_ = ([DPPH]_ Total_ / [DPPH]_0_ ) x100

 - %DPPH remaining = Value of absorbance of each concentration/Value of 0 absorbance (Solvent+DPPH).

-The results are expressed as mmol TE/g of dry extract.

IC_50_ values correspond to concentration of the extract in the reaction mixture which decrease the initial DPPH concentration to 50%. They have been analyzed using PHARM/PCS – version 4 software. The data are presented as mean values ± standard deviation. Each determination was performed in duplicate and repeated at least two times.

*Ferric reducing/antioxidant power (FRAP)*

The ferric reducing ability of the extracts under study was evaluated according to the method described by Benzie and Strain (16) with minor modifications (17). The FRAP reagent contained 10 mM of 2,4,6-tripyridyl-s-triazine (TPTZ) solution in 40 mM HCl, 20 mM FeCl_3_·6H_2_O, and acetate buffer (300 mM, pH 3.6) (1:1:10, v/v/v). 50 µL of a methanolic/water solution containing different concentrations of the extracts tested (0.06, 0.12, 0.25, 0.5, 1, and 2 mg/mL) or of the vehicle (DMSO) alone were added to 1 mL of the FRAP reagent, and the absorbance was measured at 593 nm in a spectrophotometer (Shimadzu, Japan) after incubation at 20 °C for 4 min against air. Results are expressed as mmol Fe^2+^ equiv/g dry extract. Each determination was performed in duplicate and repeated at least two times.

*Cytotoxicity with Trypan blue dye exclusion*

For each cancer cell line (Caco-2 and U937) 5×10^4^ cells/well were grown in a 48 plate in respective medium at 37 °C in the presence of 5% CO_2_ (v/v) for at least 24 h before addition of the solubilized extract (methanolic or ethyl acetate) in DMSO at different final concentrations (0, 5, 10, 15, 20, 25 µg/mL), Paclitaxel was used as positive control (0, 0.25, 0.5, 1, 2.5, 5 µg/mL). The cytotoxicity was also assessed for the PBMCs (5×10^4^ cell/well) at the same concentrations serving as control in order to compare anti-proliferative effects of both normal and tumor cells. The reading is taken every day for an incubation period of 72 h by heamocytometer using the trypan blue exclusion dye (McLean, Virginia, USA), where dead cells take the blue color enabling easily countable living cells. The IC_50_ correspond to a one half reduction of the growth compared the untreated control. Results are represented as the mean of three independent experiments (18).

*Analysis of the cell cycle:*

For the analysis of cell cycle phase distribution, U937 and CaCO-2 cells were plated at 4×10^4^ /mL in 48-well plates and left incubating for 24 h. Cells cultured in the presence of the extracts at different concentrations are subsequently collected, washed and fixed in 70% ethanol. After an incubation period of at least 2 h at 4 °C, cells were washed, treated with a solution of RNase (Sigma-Aldrich) and stained with propidium iodide DNA fluorochrome (PI, 50 mg/mL, Sigma- Aldrich) for 30 min at room temperature. The propidium iodide fluorescence was then measured by flow cytometry (FACScan, BD Biosciences, San Jose, CA, USA). A minimum of 10,000 cells were acquired per sample, and data were analyzed using the software Modfit 3. The percentage of cells in G0/G1, S and G2/M was determined from histograms of DNA content.

*Analysis of apoptosis:*

The test annexin V-FITC/AAD (BD Biosciences Pharmingen, San Diego, CA, USA) was used to detect early (Annexin^+^/AAD^-^) and late (Annexin^+^/AAD^+^) apoptosis. Annexin V has a high affinity for phosphatidyl serine, which becomes apparent in the membranes of apoptotic cells. The experiment was performed according to the manufacturer’s directions for the two lines “U937 and CaCO-2” exposed to methanol and ethyl acetate extracts obtained from *Rosmarinus officinalis* L. at different concentrations for a period of 24 h. The amount of apoptosis is both evaluated by flow cytometry (FACScan, BD Biosciences, San Jose, CA, USA) and Image stream analysis (Image Stream^x^, Seattle, USA). 

*Statistical analysis*

The IC_50 _values from antioxidant and anti-proliferative assays were calculated from dose response curves using linear regression analysis by fitting the test concentrations that gave percentage of inhibition values above and below the reference value (50%) using PHARM/PCS – version 4 Pharmacologic Calculation System based on “Manual of pharmacologic calculation with computer programs”, 2nd edition by R.J. Tallarida and R.B. Murray, Springer Verlag, New York, 1986. The results are expressed as mean values + SD from three separate experiments. The data was analyzed using Student’s t test. Differences were considered to be statistically significant from the controls at P < 0.05. 

## Results

*Chemical characterization:*

**Figure 1:**
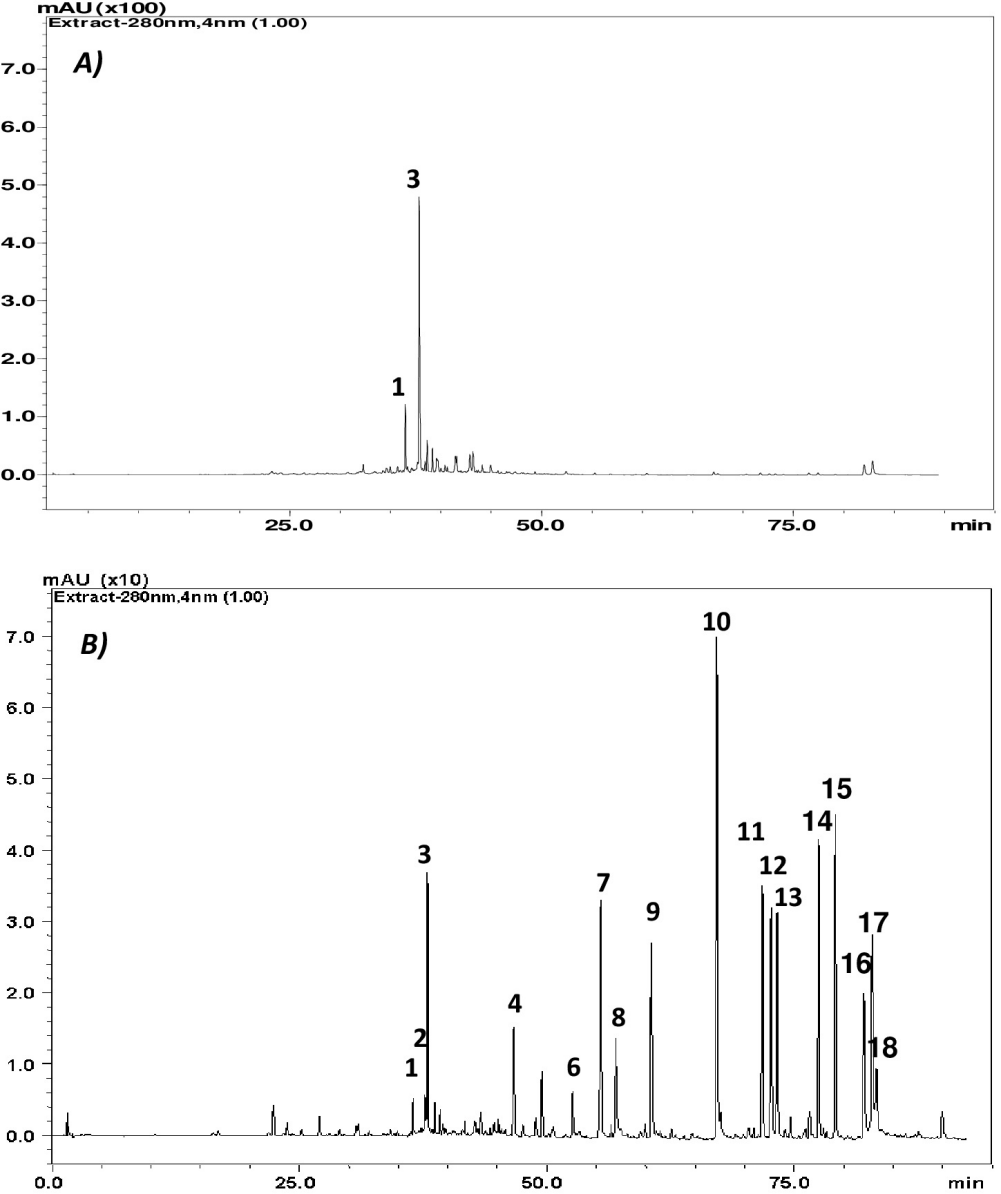
HPLC Chromatograms recorded at 280nm of *Rosmarinus officinalis *L. extracts: ***A)*** Methanolic extract, and ***B)*** Ethyl acetate extract. Only peaks corresponding to phenolic compounds or related compounds are indicated

 The total polyphenolic content was higher in Rosemary Methanolic extract (ME) with about 2.589 ± 0.0055 g/100 g of dry matter in Gallic acid equivalent compared to Ethyl acetate (EA) with 0.7637 ± 0.0056 g/100 g of dry matter. However, the latter was richer in term of diversity with about 13 identified compounds. Phenolic diterpens were mainly detected in EA, such as Carnosol, Carnosic acid and methyl Carnosate while Rosemarinic acid was major component in the ME fraction followed by the Isorhamnetin-3-O-hexoside a glycosylated flavonoid at lesser content as demonstrated in (Figure 1 and Table 1).

**Figure 2:**
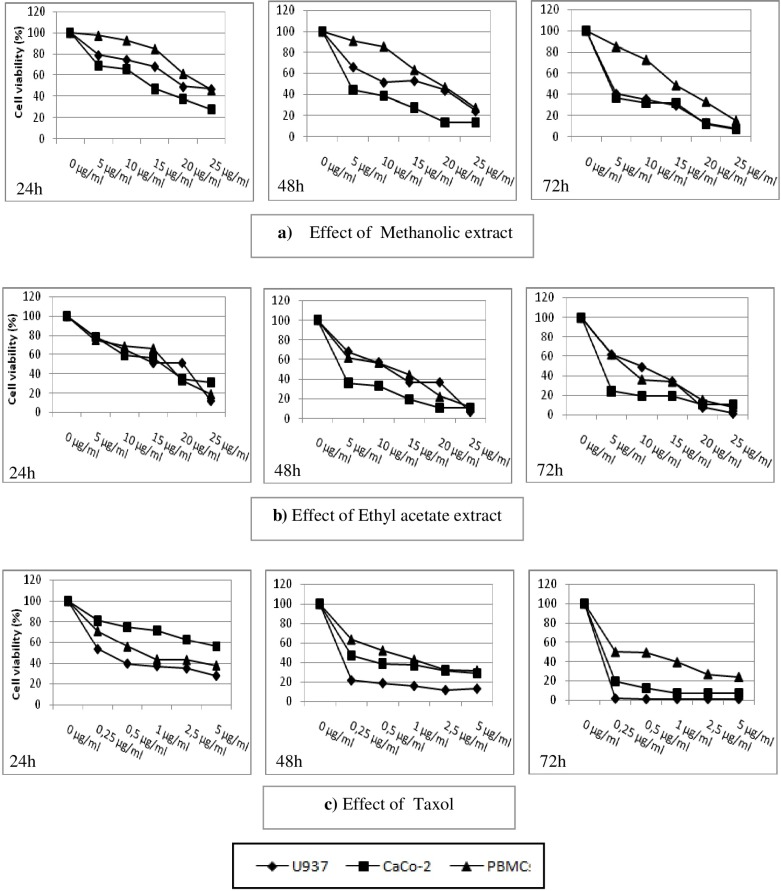
Cytotoxicity of rosemary extracts on tumor cells and PBMCs. Cells were incubated in the presence of extracts at different concentrations, and then the reading is taken for 72h using dye exclusion

**Figure 3:**
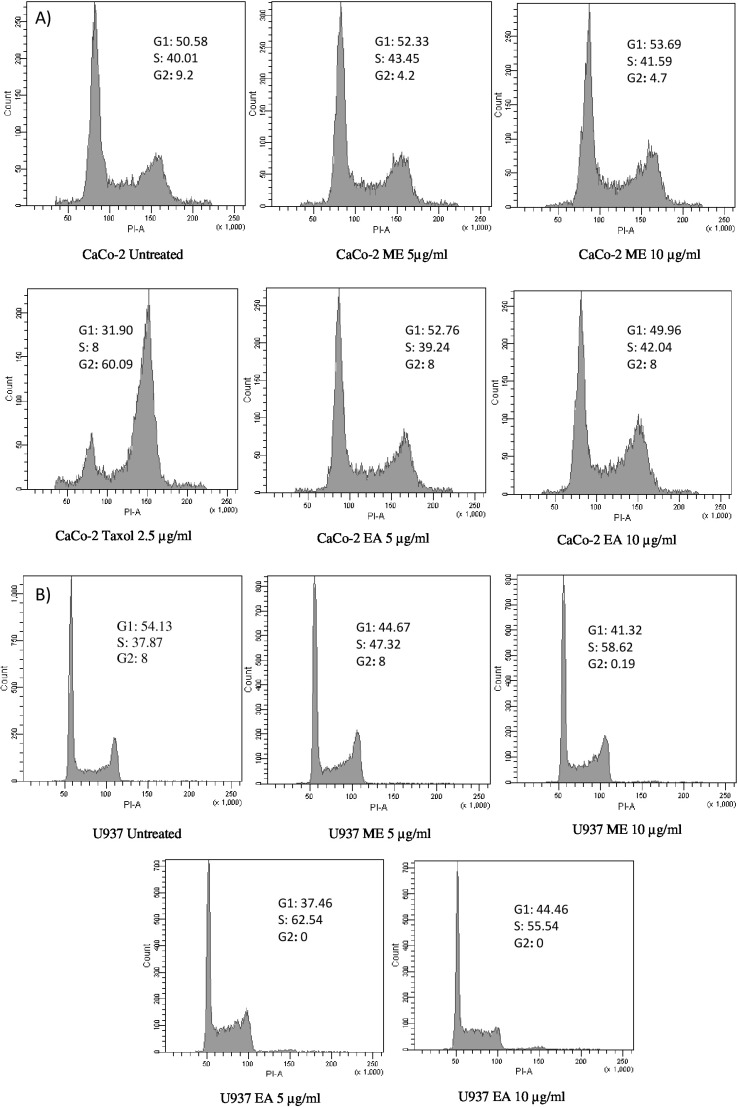
Cell cycle analysis of U937 and CaCo-2 cells treated with rosemary extracts. Cell cycle distribution in G_0_/G1, S and G_2_/M was measured after 24h of exposition to Methanolic, Ethyl acetate extracts and Taxol. Data are expressed as mean values± SD

**Figure 4:**
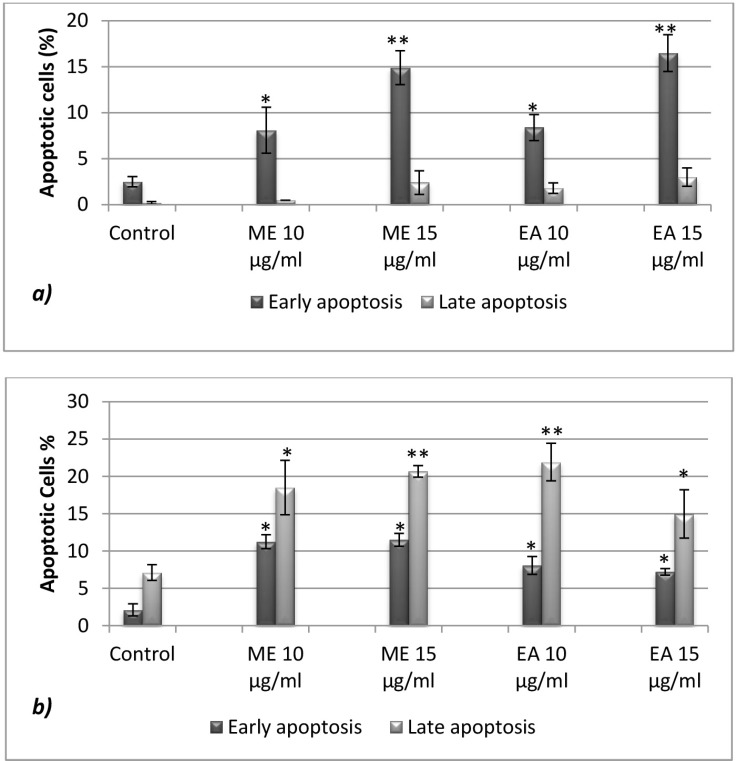
Apoptotic effect of rosemary Methanolic and Ethyl acetate extracts ***a*)** on U937 and ***b*)** CaCo-2 cells after 24h exposition. Annexin test was used to detect early and late apoptosis

**Table 1:** HPLC characterization of Rosemary Methanolic (ME) and Ethyl acetate (EA) extracts at 280 nm

**Compound**	***l*** **max**	**Retention** **time (mn)**	**%Area** **EA**	**% Area** **ME**
1. Isorhamnetin-3-O-hexoside	274, 345	36.34	0.64	10.18
2. Homoplantaginin	270, 333	37.69	1.29	
3. Rosmarinic acid	218, 328	37.80	5.91	50.11
4. NI	260, 340	37.69	1.29	
5. Scutellarein	225, 277	60.42	2.01	
6. NI	274, 345	71.44	7.06	
7. Cirsimaritin	270, 333	72.92	5.82	
8. Rosmanol	218, 328	37.69	5.91	
9. Genkwanin	218, 328	60.42	1.29	
10. Carnosol	260, 340	71.44	2.01	
11. Rosmadial	225, 277	60.42	2.01	
12. 4’Metoxytectochrysin	260, 340	71.44	7.06	
13. Rosmarinic acid methyl ester	225, 277	72.92	13.86	
14. NI	274, 345	71.44	14.18	
15. Carnosoic acid	260, 340	81.60	2.01	
16. NI	225, 277	71.44	5.82	
17. Methyl carnosate	260, 340	82.42	7.40	
18. NI	213, 278	82.89	2.27	

**Table 2:** Antioxidant and antiproliferative activities of *Rosmarinus officinalis* L extracts

**Extract PBMCs **	**DPPH** **IC**_50_** (mg/ml)**	**FRAP** **(mmol Fe**^2+^**/g)**	**U937**	**CaCo-2** **IC**_50 _**(µg/ml)**	**PBMCs **
Methanolic extract	0.510	1.714 ± 0.068 (0,444-0,5862)	19.64 ±1.36	14.62 ±2.00	25.4 ±2.54
Ethyle Acetate extract	0.719	1.240 ± 0.052 (0,3515-1,471)	14.85 ±0.20	14.95 ±2.32	14.43 ±4.33
Taxol	-	-	0.23 ±00	3.21 ±1.72	0.96 ±0.24

*Antioxidant assays*

The total phenolic content of the studied extracts was determined by Folin-Ciocalteu method. Data in Table 2. show that the highest amount of total phenols was detected in the methanolic extract with an equivalent of gallic acid up to three times higher compared to the Ethyl acetate fraction. Indeed, this higher content was positively correlated with an increased antioxidant property, in terms of DPPH radical scavenging capacity with a reduced IC_50_ of 0.510 mg/mL and an elevated trolox equivalent of 1.29 ± 0.07 mmol TE/g extract. Moreover, the methanolic extract was characterized by a higher ferric reducing ability of 1.714 mmol Fe^2+^/g extract. The observed properties could be due to Rosmarinic acid, which is a dominant component in this fraction as revealed by HPLC chromatography (Table 1).

*Anti-proliferative effect of rosemary extracts*

*Cytotoxicity assay*

As shown in Figure 2. both extracts exerted an anti-proliferative activity against the tested tumor cell lines in a time and concentration dependent manner, with lesser efficiency on PBMC compared to Taxol used here as positive control. Interestingly, the antiproliferative effect observed for the methanolic extract was more pronounced on cancer cells compared to PBMC, after 48 h of incubation. In fact, the IC_50 _was two times higher compared to U937 and up to four times with regard to CaCo-2 cells (Table 2). 

*Cell cycle analysis*

Results of cell cycle analysis showed that U937 cell line was noticeably sensitive for both tested extracts (Figure 3-B). While a significant increase in the percentage of cells in S-phase was observed, a concomitant decrease in G1 and G2/M phases has been noticed. Therefore, extracts seem to be inducers of cell cycle arrest in S-phase after 24 h of treatment. The ethyl acetate extract demonstrated more efficiency to block the cell cycle in S-phase (62% of the population in S-phase at a concentration of 5 µg/mL) with a complete abolishment of G2/M phase. However, only the Methanolic fraction displayed an effect on CaCo-2 cell cycle with a decrease of G2/M phase after 24 h of incubation (Figure 3-A). On the other hand, the positive control (Taxol) was clearly able to block the growth of CaCo-2 cell line in G2/M phase with a distribution 60% at 2.5 µg/mL. 

*Apoptotic effect*

Figure 4. shows that Rosemary extracts significantly triggered apoptosis on tested tumor cell lines, with early apoptosis dominance (Annexin V^+^ and AAD^­^) in U937 and late apoptotic effect (Annexin V^+^ and AAD^+^) on CaCo-2 cells. For the latest, up to 20.6 and 21.8% of cells were late apoptotic respectively in the presence of Methanolic (15 µg/mL) and Ethyl acetate extracts (10µg/mL). The observed effects are probably attributed to the identified major components (Figure 1 and Table 1). 

## Discussion

At the light of the obtained data, the identified compounds present in rosemary extracts can be classified into three groups: diterpenes, flavonoids and phenolic acid. The diterpenes structures were represented by carnosic, Carnosol, Rosmanol, Methyl Carnosate and Rosmadial. Flavonoids, including Genkwanin, 4’Metoxytectochrysin, Homoplantaginin, Scutellarein and Cirsimaritin, while rosmarinic was the single identified phenolic acid. These major components have been reported in many studies evaluating the composition of Rosmary extracts (19). Our results are in agreement with many studies reporting that Rosemarinic acid is the most abundant phenolic compound identified in the extracts of labiatea family (20, 21).

Several reports have demonstrated that anti-oxidative activities such as free radical scavenging, lipid peroxidation and metal chelating activities from natural extracts can enhance anti-cancer activities of many chemical anti-cancer drugs (22, 23, 8, 24). Significant correlations between antioxidant and antiproliferative activities have been established on HeLa, MCF7 and HT-29 cell line models (25, 26, 27, 28). The authors assumed that phenolic compounds or vitamin C, as strong antioxidants, might influence cell redox state leading to decreased cell proliferation. Indeed, cancer cells continuously proliferate under a state of oxidative stress because this condition increases their potential to survive by activating redox signaling that may lead to the activation of prosurvival factors such as NFkB and AP-1 and the inactivation of tumor suppressor genes such as p53 or mutations (3). Mild levels of reactive oxygen species have been shown to induce the proliferation of cancer cells (25). 

Data obtained by Del bello *et al.* (25) have revealed that H_2_O_2_ formed during the gamma-glutamyl transpeptidase (GGT) activity appears to maintain U937 cell proliferation and protect it from apoptosis, which is inhibitable by antioxidant treatments such as catalase and Trolox C. In addition, GGT inhibition affected by acivicin was able to decrease H_2_O_2_ formation and as a consequence cell proliferation; concomitantly, GGT inhibition was followed by a decrease in poly (ADP-ribose) polimerase (PARP) activity and by the onset of apoptosis**.** Methenolic extract showed higher antioxidant activity compared to Ethyl acetate fraction, probably due to its enriched content of Rosmarinic acid. In fact, it has been reported in few studies that natural extracts from labiatae family with higher content of Rosmarinic acid possess higher radical scavenging activity (21). Comparable observations were reported by Zakariat and co-workers (3) for *Melastoma malabathricum* Leaves, where the highest content of rosmarinic acid in the methanolic fraction was associated with a maximum antioxidant capacity. On the other hand, the Ethyl acetate extract activity seems to be related to its dominant diterpens **(**Carnosol, Carnosic acid and methyl Carnosate). In fact, the potent antioxidant properties of rosemary extracts (up to 90% of the total antioxidant activity) have been attributed to its phenolic diterpenes, including carnosic acid, carnosol, and rosmanol (29). Finally, the antioxidant properties displayed by rosemary extracts, specially the methanolic one, could favor their anti-tumor potentialities. 

In the present study we showed also that rosemary extracts exerted cytotoxic effects on both tested cell lines U937 and CaCo-2 with lesser activity on the peripheral blood mononuclear cells. Our results are in accordance with those reported by Cheung and Tai (30) and Cheng *et al.* (8) where the Crude ethanolic rosemary extract has shown anti-proliferative effects on human leukemia and breast carcinoma cells. The studied extracts can be classified furthermore as active (IC_50 _values 20 µg/mL) according to the National Cancer Institute (NCI), USA (31). Furthermore, our data corroborate many studies indicating a selective growth inhibitory activity of natural extracts against cancer cells with less effect on normal cells (32, 33, 34, 5). Several antiproliferative mechanisms could be implicated based on the type of phytochemical constituents present in each of the extracts, including: 1) the induction of cyclin-dependent kinase inhibitors or the Ca2þ-dependent apoptotic mechanism, 2) modulation of cell cycle arrest at the G1/S phase, 3) inhibition of the cell-survival kinase and the inﬂammatory transcription factors, or 4) the down-regulation of the antiapoptotic gene products (3). We thus investigated whether our extracts could affect cell cycle and/or display pro-apoptotic effects as a mechanism of the observed cytotoxicity.

Cell cycle analysis revealed that both extracts were able to affect U937 cell cycle distribution at S-phase level, with a concomitant decrease in G1 and G2/M phases. Results are in concordance with those reported by Vaz and co-authors who demonstrated that *Clitocybe alexandri* ethanolic extract blocked the cell cycle distribution at S-phase in a dose dependent manner (35). Similarly, Gonzalez-Sarrias *et al.* (5) reported that the phytochemicals present in maple syrup extracts inhibit the proliferation of colon cancer cells at S-phase due to decrease of expression of cyclins A and D1. On the other hand, only methanolic fraction was able to affect the growth of CaCo-2 cells by slightly decreasing the G2/M phase. Also, Sharif and co-workers (36) have reported that for *Aronia melanocarpa *Juice with a concomitant decrease in the expression of cyclin B1. Taxol used as positive control clearly blocked the cell cycle distribution of CaCo-2 cells in G2/M phase, which corroborated the observations noticed for CHO AA8 cells (37)^.^

Several studies indicate that polyphenolic compounds promote apoptosis of cancer cells by inducing a pro-oxidant response (38). However, the nature and the source of reactive oxygen species (ROS) produced in response to polyphenols remain poorly studied. A role for mitochondria-derived superoxide anions has been suggested in resveratrol- induced apoptosis in HT-29 human colorectal carcinoma cells (39) and a reduced glutathione antioxidant system in hispolon-induced apoptosis in human gastric cancer cells (36). Cheng *et al*. (8) have also demonstrated that rosmanol can potently induce apoptosis in COLO 205 cells via mitochondrial pathways, where treated cells exhibited a signiﬁcant increase of both cytochrome-c and AIF (Apoptosis-inducing factor) into the cytosol.

## Conclusion

The present study indicates that *Rosmarinus officinalis* L. extracts markedly inhibited the proliferation of two tested cancer cells, U937 and CaCo-2 in a time and concentration-dependent manner. Interestingly, the antiproliferative activity of the methanolic fraction was more pronounced on tumor cells compared to PBMCs. The growth inhibitory effect was associated with an arrest of cell cycle progression in S phase for U937 with a concomitant down-regulation of the G1 and G2/M phases. On the other hand, only Methanolic extract slightly affected CaCo-2 cell cycle with a decrease of G2/M phase after 24 h of incubation. 

Rosemary extracts displayed in addition a remarkable pro-apoptotic activity on both cell lines as indicated by annexin V labeling, with early apoptosis dominance for U937 and late apoptotic effects on CaCo-2 cells. The displayed antioxidant properties may be also in favor of the observed anti-tumor potentiality. Taken together, these results suggest that phenolic compounds may impart interesting biological effects to *Rosmarinus officinalis *L.*,* which may represent a potential source of chemopreventive agents. Dominant phenolics identified in rosemary extracts, namely diterpens and rosmarinic acid warrant to be purified for further investigations. 
